# Improving access in medical physics residency programs for physicists with disabilities

**DOI:** 10.1002/acm2.14518

**Published:** 2024-09-16

**Authors:** Jessica M. Fagerstrom, Grace Eliason, Hania Al‐Hallaq, Brian A. Taylor, Muhammad Ramish Ashraf, Natalie Viscariello

**Affiliations:** ^1^ Department of Radiation Oncology University of Washington Seattle Washington USA; ^2^ Department of Radiology University of Colorado School of Medicine Aurora Colorado USA; ^3^ Department of Radiation Oncology Emory University Atlanta Georgia USA; ^4^ Imaging Physics UT MD Anderson Cancer Center Houston Texas USA; ^5^ Department of Radiation Oncology Stanford University Palo Alto California USA; ^6^ Department of Radiation Oncology University of Alabama Birmingham Birmingham Alabama USA

**Keywords:** accessibility, accommodations, disability, education, residency, teaching

## Abstract

Within the landscape of medical physics education, residency programs are instrumental in imparting hands‐on training and experiential knowledge to early‐career physicists. Ensuring access to educational opportunities for physicists with disabilities is a legal, ethical, and pragmatic requirement for programs, considering that a significant proportion of the United States population has a disability. Grounded in conceptual frameworks of competency‐based medical education and the social model of disability, this work provides an introduction to some practical recommendations for medical physics residency programs. Strategies include embracing universal design principles, fostering partnerships with disability service offices, using inclusive language, developing and publicizing clear procedures for disclosing disabilities and requesting accommodations, and maintaining an overall commitment to equitable access to education. This work urges medical physics residency leadership to proactively move towards training environments that support the needs of residents across the spectrum of disability, highlighting why disability inclusion fundamentally enriches diversity.

## 1 INTRODUCTION

Medical physics residency programs in the United States play a pivotal role in providing training and experiential learning for the next generation of medical physicists. In pursuit of inclusivity and diversity within the healthcare workforce, it is imperative to address challenges faced by residents with disabilities at this essential step in their education. Here, we use the World Health Organization's definition of disability, which states that “disability results from the interaction between individuals with a health condition, such as cerebral palsy, Down syndrome and depression, with personal and environmental factors including negative attitudes, inaccessible transportation and public buildings, and limited social support.^1^”

In this context, biases, policies, and infrastructure represent tangible obstacles that can hinder the academic and professional success of residents with disabilities. This paper seeks to explore and advocate for best practices in medical physics residency education to foster learning environments that are not only inclusive, but actively support the diverse needs of residents. In the subsequent sections, we propose actionable recommendations to integrate into medical physics residency programs and share two groups’ experiences in the form of case studies from related areas of medical education. While it would be impossible to address all facets of this important topic comprehensively, this paper is intended to offer a brief introduction and starting point to residency programs aspiring towards accessibility for their trainees with disabilities.

## 2 MOTIVATION

Medical physics residency programs bear a responsibility to provide equitable access to quality education and professional development to all qualified physicists, including physicists with disabilities. By embracing diversity and inclusivity, programs not only promote equity but also enrich the medical physics community with a broader spectrum of perspectives and talents, ultimately driving innovation and excellence within the field. In the 2021 AAPM Equity, Diversity, and Inclusion Climate Survey, 7% of respondents indicated that they had a disability, impairment, or condition,^2^ while in the 2021 Canadian Organization of Medical Physicists (COMP) Equity, Diversity, and Inclusion Climate Survey, 23% of respondents indicated that they had either a disability or health condition.^3^ The discrepancy between these values indicates either that there is a deficit of disabled people in the medical physics workforce in the United States compared to Canada or that the AAPM survey was designed in such a way that members did not feel comfortable disclosing their disability status or were not aware that they are classified as having a disability. The employment rate for disabled Americans reached a record high of 22.5% in 2023, according to the Bureau of Labor Statistics,^4^ while Canada's Disability Inclusion Action Plan for 2022 indicated 59% of disabled Canadians were employed.^5^ Given the shortages along the pipeline – of physics undergraduates, residency programs, and medical physicists^6^ – and the growing fraction of practicing medical physicists with disabilities, inclusion is a crucial practical consideration. The obligation to provide quality training to physicists with disabilities is especially critical considering the current shortage of medical physicists,^7^ given that a significant fraction of practicing medical physicists report having disabilities.

Moreover, a commitment to providing exceptional patient care mandates that future medical physicists are equipped with the skills and insights necessary to serve the entirety of the expected patient population. Approximately 26%−28% of the adult population of the United States has a physical disability.^8^, ^9^ Healthcare workers with disabilities are well prepared to consider and provide care tailored to the diverse needs of patients with various accessibility requirements.^10^ They can also provide unique perspectives of the healthcare system and improve care by adding an empathic view of the patient experience.^11^ In addition to social justice and patient‐centered motivations for providing accessible medical physics residency education, educational programs are legally required to provide equal access to opportunities, programs, and services for individuals with disabilities through the Americans with Disabilities Act as amended (ADA‐AA).^12^ This legislation requires nondiscrimination on the basis of disability, and also requires that programs provide reasonable accommodations to facilitate access. For these reasons, medical physics residency programs should expect and be prepared to train talented physicists with disabilities.

## 3 THEORETICAL FRAMEWORK

A theoretical or conceptual framework serves as a fundamental structure that underpins the theoretical aspects of academic work, providing a roadmap for understanding and addressing complex issues.^13^ It offers researchers a systematic way to organize and conceptualize their ideas. Within the context of this work, we have adopted the competency‐based framework for access and inclusion in medical education, as proposed by Curry, Meeks, and Iezzoni.^14^ This framework encourages educational institutions to move away from technical standards‐based education that can be discriminatory toward trainees with disabilities and move toward competency‐based medical education, incorporating entrustable professional activities (EPAs). Further, our paper is rooted in the application of the social model of disability, which provides a critical perspective for understanding disability as a social construct.^15^ The social model of disability is in contrast with a medical model of disability: while the medical model positions disability as a deficit of an individual, the social model recognizes disability as the result of environmental and social barriers that inhibit full participation of individuals and groups with disabilities.

## 4 SPECTRUM OF DISABILITIES

Health professional trainees may experience a variety of disabilities. Within the framework of the social model of disability, residency programs should be familiar with the complex interplay between an individual's capabilities and the sociocultural and built environments. Disabilities within this context encompass a broad spectrum, including physical, neurological, sensory, mental, and cognitive conditions.^16^ Physical disabilities include mobility impairments, sensory impairments include blindness or hearing impairment, and chronic conditions include arthritis or muscular dystrophy. Mental disabilities encompass diverse conditions such as anxiety disorders, depression, schizophrenia, and neurodevelopmental disorders like autism spectrum disorder. Cognitive disabilities may involve memory, attention, or problem‐solving abilities. A survey of United States medical students found that the most common reported disabilities were attention‐deficit/hyperactivity disorder, learning disabilities, and psychological disabilities^17^ – note that these prevalent disabilities are sometimes classified as non‐apparent (sometimes called “hidden” or “invisible” disabilities), and that the majority of reported disabilities are non‐apparent.^18^

## 5 LEGAL CONSIDERATIONS

According to the legal framework of the ADA‐AA,^12^ employers with fifteen or more employees are required to provide reasonable accommodations to individuals who meet the definition of having a disability. While legal requirements obligate medical physics residency programs to provide reasonable accommodations for physicists with disabilities, it is important to acknowledge that cost may be a concern for some programs. Programs can argue undue hardship based on financial constraints; however, this argument is rarely successful and financial hardship is typically judged based on the resources of the parent organization such as the larger university or hospital system.^18^, ^19^, ^20^ In a case where participation in a procedure may impact trainee safety (e.g., implanted devices in an MRI setting), alternative learning methods should be pursued. A case study is presented later in this work of a radiation oncology residency's successful approach to implementing accommodations for a Deaf trainee that includes funding considerations.

Medical physics residencies with a disability services (DS) office, often available at universities, should channel all accommodation‐related communications and processes through this office. The DS office will coordinate accommodations, ensure compliance with legal requirements, and safeguard the privacy of residents’ health information. Residents should be encouraged to disclose disabilities directly to the DS office, rather than to their supervisors, to prevent unnecessary disclosure to colleagues. The DS office is then responsible for implementing necessary accommodations.

**FIGURE 1:**
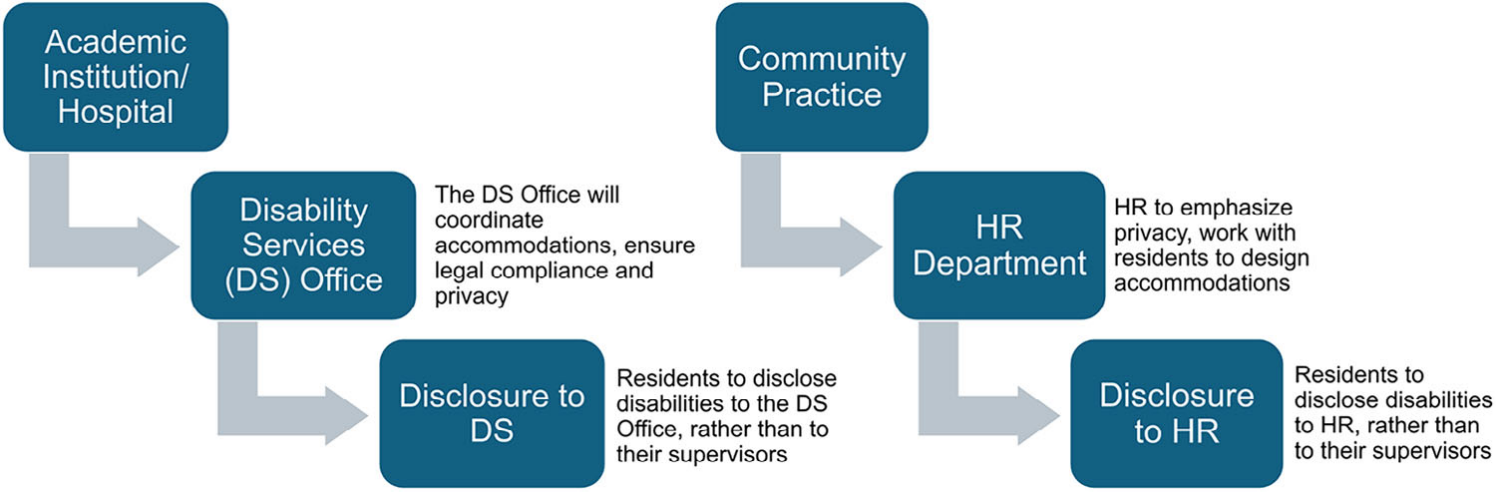
Suggested workflows for disclosure process at institutions with a disability services office available and at institutions with no disability services office, but a human resources department.

In community practice residencies in which a dedicated DS office may not exist, disclosure and accommodation procedures may be different. In such cases, especially when residents are considered employees, institutions must still adhere to all federal privacy laws. Residents should be encouraged to disclose directly to the human resources (HR) department, and the accommodations process may be handled directly through HR. In the absence of a DS office, the HR department must emphasize the importance of privacy and detail how HR will collaborate with the residency program to implement necessary accommodations. This process is illustrated in Figure 1.

In medical physics residency programs without a dedicated DS office or a formal HR department, program administrators should anticipate a greater need for direct communication and support for residents disclosing disabilities. Without access to specialized resources, administrators may need to assume a more hands‐on role in coordinating accommodations and ensuring the confidentiality of disclosure processes. In this case, it will likely not be possible to maintain as much resident privacy as ideal, but programs should still strive to maintain as much confidentiality as possible and information should be shared only with team members as absolutely necessary.

Regardless of university or community‐based setting, clear and well‐defined procedures for disclosure and accommodation requests are essential for fostering an inclusive learning environment and for ensuring privacy protection and legal compliance. It must be noted that while learning opportunities are the goal of residency training, patient and trainee safety is a top priority.

Though the majority of trainees discussed in this paper are likely classified as employees, some postdoctoral fellows may be categorized as independent contractors. Independent contractors are not guaranteed protections by anti‐discrimination laws including the ADA‐AA. To address this concern, it is recommended that programs establish clear and inclusive policies that guarantee all trainees, regardless of employment classification, have equal access to necessary accommodations.

## 6 COMPETENCY‐BASED LEARNING

Competency‐based medical education emphasizes outcomes, in contrast to emphasizing other conventional metrics, such as the length of time trainees spend on a given topic or the requirement to demonstrate specific technical abilities.^21^ In other words, competency‐based medical education focuses on the “what” instead of the “how.” A component of competency‐based education can be the use of EPAs. EPAs are responsibilities or units of work that trainees are authorized to undertake without supervision after they have demonstrated a requisite level of expertise. These activities are observable, measurable, and tailored to a specific discipline.^22^

As medical physics programs develop and assess new EPAs, it is suggested they consider access for many types of learners when finalizing language, and to use a balanced approach to accommodate all types of learners while maintaining the integrity of the standards essential for program completion. For example, Curry et al.^14^ cite two possible approaches to requirements regarding a physical examination of a patient. One approach states that candidates “should have sufficient exteroceptive sense (touch, pain, and temperature); proprioceptive sense (position, pressure, movement, stereognosis, and vibratory); and motor function to carry out the requirements of the physical examination,” contrasted with the Association of American Medical Colleges’ (AAMC) report on EPAs for entering residency, which states that candidates must be able to “perform a clinically relevant, appropriately thorough physical exam pertinent to the setting and purpose of the patient visit.” Both of these requirements, as written, aim to outline standards for conducting a physical exam; however, the latter language accommodates the natural differences in students’ needs.

Currently, there do not yet exist consensus sets of EPAs for medical physics residencies, but one possible example of an EPA may involve routine assessment of computed tomography dose indices (CTDIs) and image quality of a CT unit. Aspects of the EPA may be written in technical language that specifically require the resident to lift, position, and manipulate standard 16‐ and 32‐cm acrylic CTDI phantoms prior to selecting relevant scanning protocols, acquiring charge measurements, analyzing images, and identifying and troubleshooting problems that arise. A more accessible version of this EPA may be to require the resident to demonstrate the correct use and positioning of the phantom and auxiliary equipment prior to acquiring and analyzing data. This more general language still demands the resident understand the required positioning of the equipment, but allows the resident the option to direct an able‐bodied assistant to position the phantom under the resident's direction.

## 7 DISCLOSURE AND ACCOMMODATION PROCESSES

Disability disclosure to an employer or potential employer can be a difficult topic to navigate, particularly during the Medical Physics Residency Match. The United States Equal Employment Opportunity Commission enforces federal laws that prohibit discrimination against applicants or employees because of a person's race, color, religion, sex (including pregnancy, gender, and sexual orientation), national origin, age, disability, or genetic information.^23^ Though, to our knowledge, no publications currently exist for medical physics trainees’ experience with disclosure and accommodations, there is some survey‐based research on the physician resident experience. This work has found that a significant number of trainees requiring accommodations do not request them.^24^ Resident physicians with disabilities cite perceived bias or stigma, as well as lack of clear processes for disclosure as reasons for not requesting accommodations.^25^ A previous medical physics education‐focused study noted that any question that could potentially lead to discrimination against a candidate, or perceived as such, should not be included in the residency interview process.^26^ Residency candidates with disabilities may be hesitant to disclose needed accommodations during the recruitment, application, and interview processes leading up to match, stemming from concerns that disclosure may negatively affect their chances of securing desired interviews or programs ranking them.

To address these concerns, programs can proactively discuss general policies and procedures with candidates. As examples, the Docs with Disabilities Initiative suggests sample language before the interview: “We welcome qualified applicants with disabilities who can perform the essential functions of the training program with or without reasonable accommodations. If you are a trainee who needs accommodation during your interview, please contact…” Following the match, they suggest sample language in communications with matched trainees: “We aim to create a fully accessible environment for residents with disabilities who require accommodations to meet the essential functions of our program. Get in touch with our Human Resources office to get started. We value disability as a form of diversity in our program. We engage in a robust process to determine reasonable workplace accommodations.^27^” During recruitment, interviews, and matching, program leadership should connect with their institution's HR office, and if available, their DS office, and ensure that program leadership and administrative staff understand the process for disclosure and how to respond to disclosure.^28^ The ADA specialist should coordinate with the residency program director, noting that accommodations should be developed by a person who does not directly supervise the resident requiring accommodations in a way that protects the resident's privacy.

**FIGURE 2:**
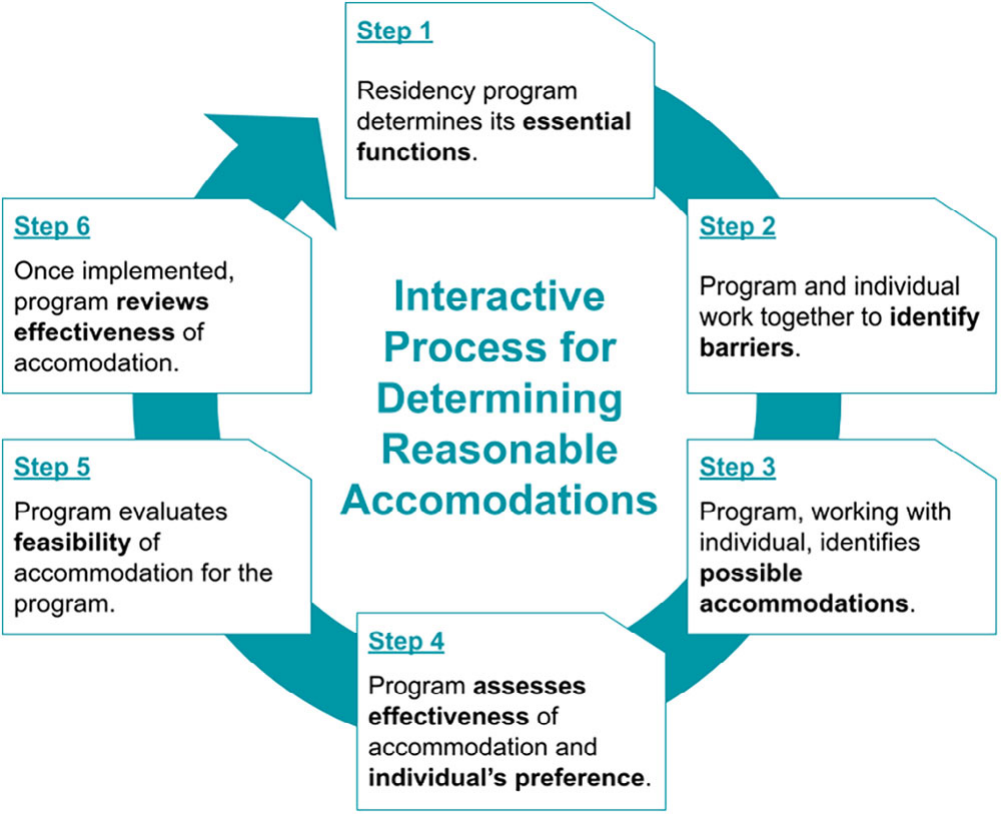
The AAMC's interactive process of determining reasonable accommodations for residents who are disabled.^18^ If ineffective, the program should enter back into the interactive process to review potential alternative accommodations. Reproduced with permission from the Association of American Medical Colleges.

Maintaining clear and well‐publicized disability‐related policies and resources helps to ensure that programs effectively support residents requiring accommodations. These policies should describe key steps for the accommodations process. According to the AAMC, the process to determine reasonable accommodations for students and trainees with documented disabilities is both interactive and iterative, and includes the consideration of patient safety throughout. This process is illustrated in Figure 2.

## 8 PRACTICAL STRATEGIES FOR ACCESSIBILITY

Accessibility in residency is an ongoing process that extends far beyond the recruitment and matching process and even beyond implementation of accommodations. Because necessary accommodations are unique to each trainee, it is not possible to detail here all possible options. Instead, educators are encouraged to adopt the principle of “universal design,” which seeks to establish an inclusive and supportive learning environment for *all* learners. A quintessential example of universal design is the “curb cut.” Curb cuts are designed to assist wheelchair users but have proven beneficial to a much broader range of pedestrians, including those pushing strollers, luggage, or bicycles. By making sidewalks accessible for wheelchair users, many people benefit. Similarly, embracing universal design principles in residency education involves implementing strategies that accommodate specific needs, with the advantage of enriching the learning experience for the entire cohort, as well as staff, patients, and future trainees.^29^

Practical ideas include incorporating universal design principles into both the learning environment as well as the curricula. Educators can present content and offer formative assessment options in multiple formats.^30^ They can intentionally add visual representations of data, such as graphics and charts, to provide clear references for learners with varied learning preferences, and offer learners prompts and questions ahead of a scheduled discussion to empower learners to prepare at their own pace. As an example, when considering overarching restrictions such as prohibiting students from using electronic devices in lecture or clinic in order to limit distractions, educators can evaluate in advance if the practice may be exclusionary for some students. Granting individual allowances for students who require electronic devices as an accommodation while the rest of the class cannot use devices may make the student requiring the accommodation feel alienated or resented by their fellow students. Instead, an educator may require that all electronics be set to “airplane mode,” allowing all students the benefit of devices while minimizing their distractions.^31^ Similarly, traditional paging systems may present barriers for Deaf or low‐vision residents taking on “physicist of the day” duties. One way programs may choose to address this limitation could be to make other options available, such as tactile/vibrating pagers.^18^ Finally, residency can be mentally, physically, and emotionally demanding. Protected time for health appointments is a key component of accessible training programs, acknowledging the importance of prioritizing health of trainees during a challenging period.^32^ Because all members of the healthcare team require medical appointments, prioritizing time for healthcare is an important aspect of universal design.

One important consideration for training educators revolves around language choice when discussing disability. Person‐first language (e.g., “person with a disability” or “physicist with a disability”) is used to emphasize the individual first and their disability second. Identity‐first language (e.g., “disabled person” or “disabled physicist”) acknowledges disability as an inherent part of identity, and is preferred by many individuals.^33^ At the same time, some individuals may not associate their identity with their disability and may want to be addressed just as a person or colleague especially when disability is not in the context of the discussion. Because preferences are personal, it is best to confirm a person's preferred language directly with each individual. If it is not possible to confirm language preference in advance, such as when writing language about accessibility policies for a program webpage, it is recommended to default to person‐first language and update it if informed otherwise.^34^ When referring to diversity, equity, and inclusion efforts, explicitly include disability in official language.

## 9 BENEFICIAL FOR ALL

Implementing changes to a program with the universal design ideal in mind requires resources. Providing training for faculty and staff is crucial for fostering a collective awareness and commitment to accessibility. While implementation of universal design principles in medical physics residency education may require financial investment, dedicated effort for training, and allocation of various resources, it pays dividends in creating an inclusive and accessible learning environment. The commitment to universal design ensures that individuals with diverse abilities can participate in the learning process, but it also enriches the educational experience for all involved. A key aspect of universal design is its broad applicability. Environments designed to be accessible for trainees with varied accessibility needs are helpful not only to trainees, but also to faculty, staff, and patients, who may also have a diverse array of accessibility requirements. For example, able‐bodied faculty/staff may undergo surgical procedures of extremities that would require accommodations. In addition, the need for accommodations may evolve over the span of one's career (e.g., vision or hearing decline with age). Working in an accommodating environment can lessen anxiety related to such life events and improve overall workplace wellness.

It should be noted that trainees with disabilities who have successfully navigated the educational path to enter a medical physics residency often possess professional strengths that are correlated with success. These may include time‐management skills, self‐awareness, self‐determination, and resilience.^35^ Such skills are cited as desired in an incoming resident by program directors, as they contribute to successful completion of the residency program^36^ and are important for medical physicists to thrive in a clinical setting.^37^

## 10 CASE STUDY EXAMPLE 1: RADIATION ONCOLOGY RESIDENT EXPERIENCE

A radiation oncology residency at Johns Hopkins University as described by Hill et al.^38^ serves as an example of successful implementation of accommodations in a professional medical residency program The residency program supported a Deaf resident who used American Sign Language (ASL). In this section, we use identity‐first language based on the paper's practice, with the capitalized word “Deaf” referring to a community and culture, and with lowercase “deaf” referring to the condition of not hearing.

The radiation oncology residency program prioritized collaboration with the trainee throughout the process of developing accommodations, aiming to provide the individual's required and preferred accommodations while noting the importance of tailoring their response to an individual's needs (i.e., acknowledging that other Deaf trainees may have different communication requirements). Faculty and staff were trained to educate themselves on working with Deaf or hearing‐impaired colleagues and to be prepared to address patient questions. The program engaged departmental and institutional leadership to raise awareness of the value of learners with disabilities. They also negotiated contracts with qualified ASL interpreting agencies and made adjustments to the physical workspace. These physical space changes included removing cubicle shelving and adjusting seating arrangements to allow for line‐of‐sight between co‐residents and the ASL interpreter, so that all trainees could communicate while at their desks. The resident required ASL interpretation for all clinical and didactic encounters during clinic and on‐call hours, for an average of 40−60 h per week of ASL interpretation coverage. In their paper, Hill et al. were candid about the administrative and funding requirements for the required accommodations. To address the required costs, they collaborated with their larger institution to create a financial plan, though they recommended that in the future there be a more centralized funding source. Their work highlights the importance of an individualized approach to accommodations.

## 11 CASE STUDY EXAMPLE 2: RADIOLOGY RESIDENT EXPERIENCE

A second case study details the career path of a gastroenterology fellow, who injured his spinal cord while still completing his fellowship, resulting in a physical disability limiting his hand function and requiring the use of a wheelchair.^39^, ^40^ The physician began exploring other specialties, with the goal to practice independently. He determined that diagnostic radiology was a specialty in which he would be able to perform essential functions without assistance. This was a significant career change, requiring him to complete an entirely new residency over another 4 years. The program director at Stanford University worked directly with the resident to adjust the structure of the program to meet all residency requirements and thoroughly prepare the trainee for independent practice, while also making sure that the required experiences were accessible to him.

Compatible with ACGME standards, the resident was not required to complete procedures, but was required to be able to describe how to perform them as well as be able to identify their indications, contraindications, and complications. The physician details his experience with the program working through the call schedule with him. Generally, his program required residents to complete approximately 6 weeks per year of night float, with shifts starting at 6:00PM and ending at 8:00AM, which was incompatible with the resident's medication and personal care schedule. The program instead arranged for the resident to work 5:00PM to 10:00PM weekly instead of completing 6 weeks of night float per year. This allowed the other resident on call to begin their shift at 10:00PM (shortening their shift by 4 h, which was a welcome reprieve), while still requiring the resident with the weekly schedule to complete the same number of night float hours. The resident used dictation software, and reviewed the same number of cases as his peers.

By implementing inclusive strategies, the residency programs detailed in these case studies worked to create inclusive environments for their trainees. The program's efforts met ADA‐AA requirements, but beyond legal compliance, the program made a conscientious effort to create an inclusive educational environment. These case studies are examples of two residency programs’ actions to increase the recruitment and training of healthcare professionals from underrepresented groups and to promote accessible education.

## 12 KEY POINTS AND ACTIONABLE RECOMMENDATIONS

- There are concrete benefits of diversity and inclusion in healthcare, and medical physicists with disabilities are equipped to provide care tailored to the diverse needs of patients.
- The social model of disability recognizes disability as the result of environmental and social barriers that inhibit full participation of individuals and groups with disabilities.
- There is a growing need to provide high quality education to medical physics trainees with disabilities (including non‐apparent disabilities), which includes designing individualized accommodations and fostering an inclusive environment.
- Residencies can design accessible EPAs in the context of competency‐based learning to work towards an accessible environment for their trainees.
- Employers with fifteen or more employees are required to provide reasonable accommodations to individuals who meet the definition of having a disability.
- Residents should be encouraged to disclose disabilities directly to the Disability Services Office rather than to their supervisors.
- Residency programs can proactively discuss general accessibility policies and procedures with candidates during the recruitment, application, and interview processes leading up to match.
- Educators are encouraged to adopt the principle of universal design, which seeks to establish an inclusive and supportive learning environment for all learners. A high proportion of the workforce will experience a disability at some point in their lives, and accessible environments benefit more than just individuals who identify as disabled.
- If it is not possible to confirm language preference in advance, it is recommended to default to person‐first language and update it if informed otherwise.

## 13 CONCLUSION

In conclusion, it is crucial for medical physics residency programs to prioritize inclusivity and diversity by addressing challenges faced by residents with disabilities. Embracing diversity and inclusivity in residency programs not only satisfies legal requirements, but it also promotes equity and enriches the medical physics community with a wide spectrum of perspectives and talents. By providing equitable access to quality education and professional development opportunities to all qualified physicists, including those with disabilities, residency programs encourage excellence in the field. Clear, well‐defined, and well‐publicized disability‐related policies and procedures, including those involving disclosure and accommodation requests, are crucial to ensure all residents can succeed. By adopting a competency‐based approach to education, including designing accessible EPAs, residency programs can simultaneously demand high standards of clinical care from their trainees while fostering an inclusive educational environment. While it would not be possible to outline every potential accommodation due to their individualized nature, residency programs can prioritize the concept of universal design to ensure accessibility for physicists with disabilities.

## AUTHOR CONTRIBUTION

All authors were responsible for conceptualization, methodology, investigation, resources, and writing (review and editing). The first author was also responsible for writing (original draft), supervision, and project administration.

## CONFLICT OF INTEREST STATEMENT

The authors declare no conflicts of interest.
